# Music Breathing as an innovative approach to stress management: A case study

**DOI:** 10.1192/j.eurpsy.2026.11721

**Published:** 2026-07-31

**Authors:** S. Yfantis, E. Papanikolaou, P. Alexopoulos

**Affiliations:** 1 Department of Psychiatry, General University Hospital of Patras, Patras; 2 SONORA: Multidisciplinary Organisation for Music Therapy and Research, Athens, Greece

## Abstract

**Introduction:**

Music Breathing (MB) is a receptive music therapy (MT) method by psychiatrist Dag Körlin, for stressor-related and anxiety disorders, linked to autonomic nervous system (ANS) dysregulation and hypo-/ hyperarousal symptoms. It aims to expand the window of tolerance (WoT) and restore the sympathetic/ parasympathetic nervous system balance through music listening in expanded state of awareness and mindful breathing. Clients focus on the form of the breath, like shape and frequency, and the content of the experience, like memories and emotions, express them in a (mandala) drawing, and process the experience for integration. (Körlin D. In: Grocke D, ed. Guided Imagery and Music: The Bonny Method and Beyond. 2nd ed. Barcelona Publishers 2019; 531-560)

**Objectives:**

The objective is to present a case on managing stress-related symptoms through MB sessions.

**Methods:**

The client is a 26-year-old female, experiencing hyperarousal symptoms, including intrusive thoughts, anxiety and anger bursts, restlessness, sleep disorders, headaches, and analgesic overuse. Having received MT before, we tried an innovative approach through MB: 10 hourly sessions over a 5-month period, combined with home practice. We started with a Discovery Breathing session (DB), observing normal breathing in silence, followed by Silent Breathing sessions, encouraging diaphragmatic breathing. Subsequent sessions integrated breathing modulation with music - initially with grounding qualities, in MB for Grounding (MBG), and later, in MB for Modulation - Working (MBW), more challenging music was used.

**Results:**

In the DB session (Fig. 1 – title: “Buzz”), stressful thoughts and discomfort emerged, which she portrayed as a gray film. The yellow–green butterfly was a hopeful figure. In the 4th (MBG) session, a childhood memory emerged (Fig. 2 – title: “Child memories”), bringing positive feelings of nostalgia and childlikeness. In the 8th (MBW) session, stressful imagery reappeared, represented as gray rocks (Fig. 3 – title: “Where there is darkness, there is light”). She focused on the anchor - representing her diaphragmatic breath in that moment - which helped her process the experience without ANS dysregulation symptoms. She explained that the session title reflected her awareness that, although stress was still present in her daily life, the stress-related symptoms had remitted, and she was coping more effectively.

**Image 1:**
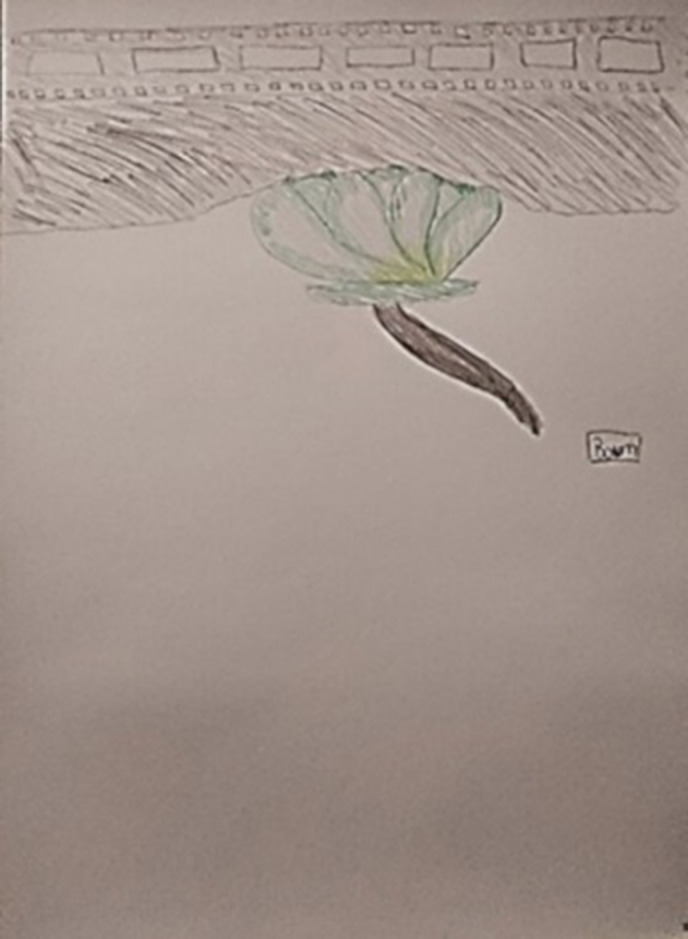

**Image 2:**
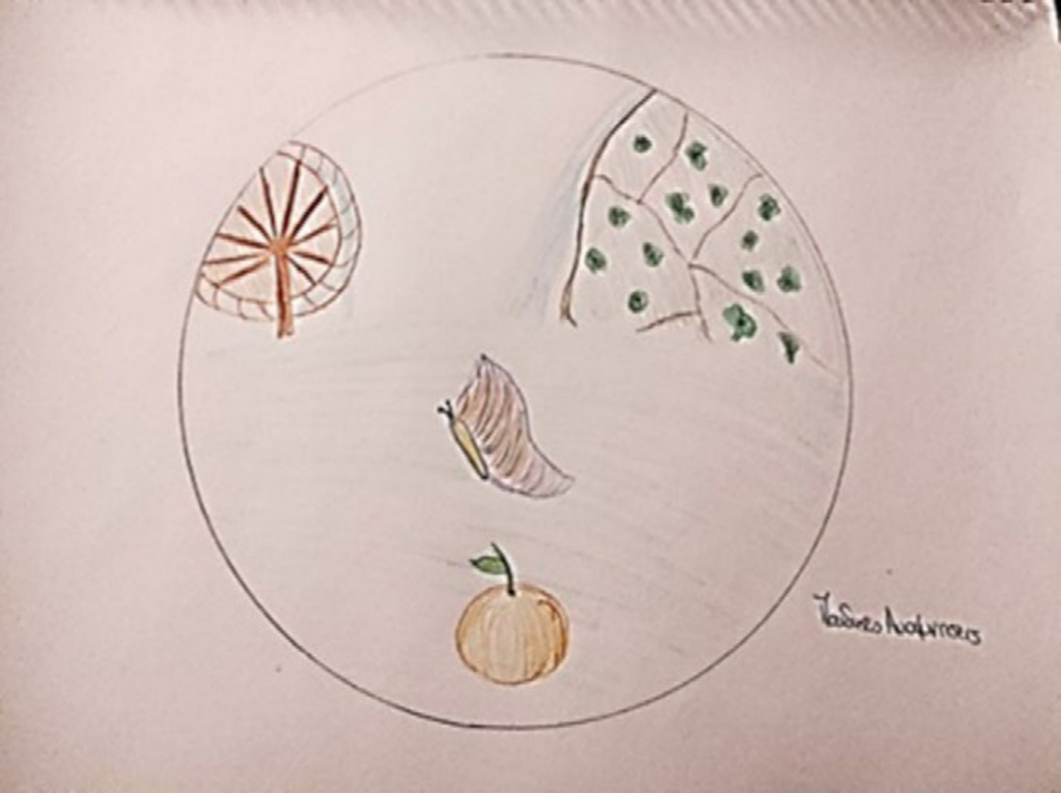

**Image 3:**
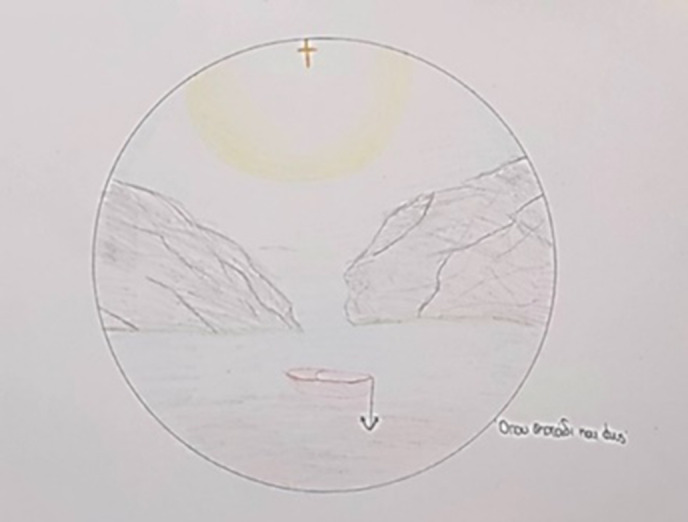

**Conclusions:**

The MB method seems to be a promising intervention for the management of stress-related symptoms, rebalancing the ANS and expanding the WoT, thus improving the coping mechanisms against stress. Stressful experiences and thoughts can be revealed and discussed, promoting more creative stress management, and supporting the process of salutogenesis. As a new and emerging method, broader use in psychology and psychiatry could encourage further research into its role in stress and anxiety management and uncover additional applications.

**Disclosure of Interest:**

None Declared

